# Her2 positive metastatic breast cancer treated with low dose lapatinib in a resource-constrained setting in South India: a retrospective audit

**DOI:** 10.3332/ecancer.2024.1758

**Published:** 2024-09-06

**Authors:** Sherin P Mathew, Manuprasad Avaronnan, Nandini Devi, V.P Praveen Kumar Shenoy

**Affiliations:** 1 Department of Medical Oncology, Regional Cancer Centre, Trivandrum, Kerala 695011, India; 2 Department of Medical Oncology, Aster MIMS, Kannur, Kerala 670621, India; 3 Department of Clinical Hematology and Medical Oncology, Malabar Cancer Centre, Thalassery, Kerala 670103, India

**Keywords:** metastatic breast cancer, her 2 positive, lapatinib, low dose, financial toxicity

## Abstract

Despite the development of newer anti-Her2 agents, access to these medicines is still restricted with lapatinib being widely used as a second-line agent in Her2-positive metastatic breast cancer. However, lapatinib at approved doses of 1,250 to 1,500 mg/day contributes to a high pill burden and financial toxicity. In a population that has an average national per capita income of only USD 2238.1, lapatinib alone contributes to a financial burden of USD 6153.56 per year (approximately USD 500 per month). A concept of ‘value meal’ has been suggested – the higher bioavailability of lapatinib with the meal being exploited to reduce its administered dose. This concept was utilised in a resource-constrained tertiary care center in South India and we report the outcomes. In our institution, consecutive patients with Her2 positive metastatic breast cancer from 1 January 2014 to 31 December 2020 who could not afford trastuzumab, lapatinib or any other anti-Her2 agent were offered low-dose lapatinib, 500 mg daily with meal. We conducted a retrospective cohort study of the safety and efficacy of this regimen. Among the 47 patients who received low-dose lapatinib, the majority had de novo metastatic disease (57.4%) and multiple visceral metastases (48.9%). The median number of lines of treatment before lapatinib was one. The disease control rate with lapatinib was 61.7%. The median progression-free survival was 7 months (95% CI: 5.6–8.4 months). The median duration of response was 4.5 months, ranging from 1.3 to 45.8 months. Only eleven patients (23.4%) experienced toxicity, mainly dermatological, with grade 3 in only one (2.1%) and no grade 4 toxicities. Low-dose lapatinib is a regimen that offers an acceptable disease control rate. This strategy requires further exploration, particularly for the benefit of resource-limited areas.

## Background

Breast cancer is the leading cause of cancer deaths worldwide and has become the leading cause of cancer deaths in India as well. It is now the most prevalent cancer in India according to the 2020 Globocan data [1–3]. 

Lapatinib is an orally administered tyrosine kinase inhibitor of both ErbB1 and Her2 receptors used in Her2 over-expressing metastatic breast cancer now recommended in the fourth line and beyond [4, 5]. Real world data show reduced utilisation of anti-Her2 agents; with nearly two-thirds not receiving even trastuzumab as an adjuvant in low middle income countries (LMICs); and less than half receiving it as the first line in metastatic disease due to affordability issues. In India, cancer treatment leads to a considerable out-of-pocket expenditure, with patients resorting to distressed means for funding [6]. Here, lapatinib is still widely used as the second line due to limited access to better agents such as Trastuzumab emtansine, Trastuzumab deruxtecan and Tucatinib [7–9].

Lapatinib is approved at a dosing of 1,250 mg/day (5 tablets) in combination with capecitabine and at a dose of 1,500 mg/day (6 tablets) along with letrozole to be taken once daily on an empty stomach [10]. The high pill burden and inconvenience of dosing while in combination with capecitabine (which is had with food), hinder compliance [11]. However, the major issue is financial toxicity. The prices of generic lapatinib in the Indian market range from INR 83 to INR 466, with a mean INR 250, per 250 mg tablet [12]. Hence, despite the use of generics, there is a considerable financial burden of approximately INR 38,020.83 per month equivalent to USD 512.80 at 2021 purchasing power parity, in a population that has an average national per capita income of only USD 2238.10 [13].

In a phase I dosing study of lapatinib, it was found that the bioavailability of lapatinib increased when administered with a low-fat meal rather than in the fasted state, and when given twice daily [14]. In a study on the pharmacokinetics of lapatinib in each prandial state, the area under the curve of lapatinib was increased 2.67-fold (167%) with low-fat breakfast and 4.25-fold (325%) with high-fat breakfast, when compared to the fasted state [11]. 

Hence the concept of a ‘value meal’ has been suggested, where 500 mg of lapatinib would be dosed with a meal instead of the recommended fasting dose [15]. The economic implications of this low-dose strategy are huge in terms of money saved per year, bringing down the expenditure by 60%, to INR 15,208.3 per month (USD 205.12).

In our tertiary care cancer institute, most patients are unable to afford lapatinib or any other anti-Her2 agents after progression on Trastuzumab. Due to financial constrains such patients receive only multiple lines of chemotherapy or hormonal agents without any anti-Her2 agent in subsequent lines. In these patients, after discussion with the patients and families, we used lapatinib at a lower dose of 500 mg/day (2 tablets of 250 mg lapatinib) with meal in combination with chemotherapy or hormones. Herein, we report our experience with the use of this regimen.

## Methods

This was a retrospective observational study among patients with Her2 positive metastatic breast cancer on treatment with Lapatinib from 1 January 1 2014 to 31 December 2020 at a tertiary cancer care center in Kerala, India. The case records were retrieved using the unique hospital identification number of patients on Lapatinib obtained from the pharmacy.

The inclusion criteria were:

1. Patients who had pathologically proven Her2-positive metastatic breast cancer.
2. Those who received low-dose lapatinib at 500 mg/day with a meal for at least 3 consecutive months or till first imaging for response assessment.
3. Such patients should not have afforded the recommended dose of Lapatinib or Trastuzumab or any other anti-Her2 agent.

The exclusion criterion was:

1. Those patients for whom the data were incomplete.

The operational definitions are as follows:

Disease control rate: Percentage of patients who showed partial or complete response (CR/PR) or stable disease (SD) while on therapy for at least 3 months.

Duration of response: Time from confirmation of CR, PR or SD until disease progression (PD).

Progression-free survival (PFS): Length of time from the start of treatment with Lapatinib until progression or death from any cause or date of last follow up.

Overall survival (OS): Length of time from the start of treatment to death from any cause or date of last follow up.

Patients with HER2-positive metastatic breast cancer were prescribed a low dose of lapatinib. They were advised to take 500 mg preferably with their noon meal and to incorporate ghee or oil-based foods, such as fried fish, into their diet.

The demographic data, the pathological characteristics of the tumour, sites of the disease, the number of lines of treatment and the agent used in combination with lapatinib were collected. The date of start of low-dose lapatinib treatment, date of first response assessment, date of progression as documented by imaging and clinical findings, date of the last follow-up, or date of death were documented for statistical analysis. All responses were documented by contrast-enhanced computed tomography of the chest, abdomen and pelvis after 3 months of the start of therapy and on clinical progression while other imaging like magnetic resonance imaging of the brain and bone scan were done as per clinical judgment. The clinical notes on toxicity with their respective CTCAE (version 5.0) grading were also recorded.

Descriptive statistics were used wherever appropriate. The survival estimates were done using the Kaplan-Meier method. The study was done following good clinical practice guidelines. A waiver of consent was obtained from the institutional research board (1616/IRB-SRC/13/MCC/10-09-2021/2) as this was a retrospective study.

## Results

After screening the records of 100 patients who received Lapatinib during the study period, 47 patients were found to satisfy the inclusion criteria of the study. The rest were screened out as they were on a standard dose of Lapatinib (*n* = 50), or because they had been on the low dose of Lapatinib for less than 3 months due to affordability issues leading to non-compliance (*n* = 3).

### Baseline characteristics

All the study subjects were females, with a mean age of 51.5 years (standard deviation, sd = 9.2). The baseline characteristics are given in the table below (Table 1). The comorbidities were predominantly diabetes mellitus and systemic hypertension. There was one patient who had metachronous malignancy – she developed metastatic breast cancer while on follow-up after treatment for low-grade non-muscle invasive bladder cancer.

The sites of metastases are as given in Table 1, of which a single visceral site was lung in 11 (23.4%); liver in 2 (8.7%); pleura in one, and brain in one patient. Among patients with multi-visceral disease, the predominant sites were lung and liver with only one patient having brain metastases.

All patients who had exclusive bone metastases had hormone receptor-positive tumours, except for one patient.

The previous treatment details are given in Table 1. Ten patients had no prior exposure to Trastuzumab before Lapatinib. The median number of lines of treatment before Lapatinib was one. Lapatinib was given in combination, mainly with Capecitabine but four patients (8.5%) received it as a single agent due to poor performance status (Table 2).

### Response

The disease control rate with Lapatinib was 61.7%, out of whom 4.3% (*n* = 2) experienced complete remission and 31.9% (*n* = 15) achieved a PR (Table 3). One patient who was clinically doing well on 2 months of lapatinib had not done imaging for confirmation of response, hence the response was unknown. The two patients who achieved complete remission had estrogen receptor (ER) positive tumours, had prior trastuzumab exposure and were on lapatinib with hormonal therapy. One of them had nodal metastases alone and was given lapatinib with exemestane as the third line, while the other had de-novo multiple visceral metastases in the lung and liver and was on lapatinib with tamoxifen as second line after progression on taxane with trastuzumab therapy. The latter patient continued to be in remission with the longest duration of response. Among 12 patients who received low-dose lapatinib and were HR negative, 5 (41.7%) had a response while 12 had a response among 35 (34.3%) with HR-positive disease. The median duration of response among HR-negative patients who received low-dose lapatinib was 6.0 months, while it was 5.9 months among those with HR-positive disease.

### Survival

**Figure 1.**
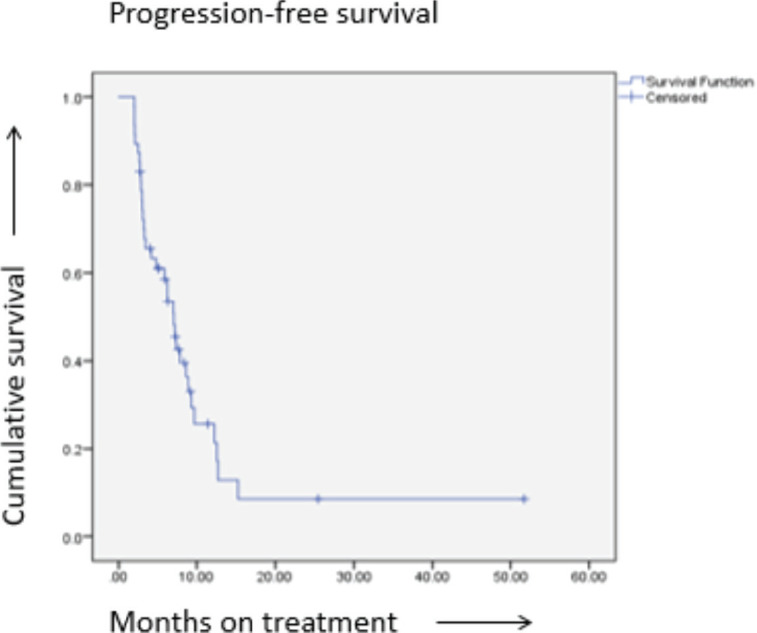
PFS.

The median follow up of the study group was 7.8 months, The median PFS was 7 months (95% CI: 5.6–8.4 months) and the median OS was 12.5 months (95% CI: 9.7–15.3 months) of the entire study group (Table 3 and Figure 1).

Six patients had a PFS of more than 12 months. Two patients continued to be progression-free for more than 24 months, the longest being 51.7 months. Both these patients had ER-positive tumours and were on Lapatinib in combination with tamoxifen as second-line therapy. Among these, one had multiple visceral metastases, in CR as previously described and the second had lung metastases. Five patients progressed within 2 months before the scheduled response assessment. Twenty nine (61.7%) patients showed clinical benefit (CR/PR/SD) at first assessment at 3 months.

The median duration of response was 4.5 months, ranging from 1.3 to 45.8 months (Table 3).

**Figure 2.**
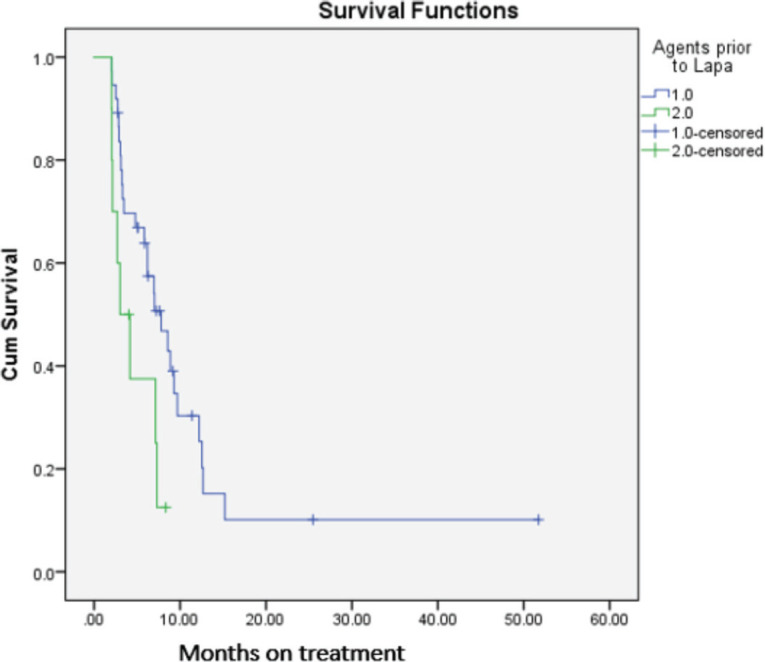
PFS of those exposed to trastuzumab (1.0 – blue) versus not exposed (2.0 – green).

On comparison of PFS between the groups with and without prior exposure to Trastuzumab, it was found that those patients with prior Trastuzumab fared significantly better than the rest, with PFS of 11.8 months (95%CI: 6.1–17.4 months) versus 4.6 months (95% CI: 3.0–6.1 months) which was significant (*p* = 0.029) (Figure 2).

There was no difference in the OS among the ER positive and ER negative subgroups. However, as the numbers are too small in each subgroup, no meaningful conclusions could be made.

### Toxicity

The low-dose lapatinib was well-tolerated. Eleven patients (23.4%) experienced some toxicity, which was predominantly dermatological (*n* = 8), grade 2 in patients who were on lapatinib and capecitabine, necessitating temporary withholding of the drugs in 3 (6.4%) patients. Grade 3 toxicity was seen only in one patient (2.1%), as palmar-plantar dysesthesia, while on lapatinib with capecitabine. Other than palmar-plantar dysesthesia, the other skin toxicities were generalised pruritus, acneiform eruptions and hyper-pigmented maculopapular rashes. One (2.1%) patient, who was on lapatinib in combination with letrozole, experienced grade 2 depressive and psychosomatic complaints, and hence, both were discontinued. Interestingly, only one patient experienced diarrhea which was only grade 2. There were no grade 4 toxicities and no cardiac or hepatic toxicities (Table 4).

## Discussion

This study needs to be seen in light of the fact that in the low-middle income countries, the only anti-HER2 agents that many patients receive continue to be biosimilar trastuzumab and generic lapatinib [16, 17]. In our study low dose lapatinib had a disease control rate of 61.7%, median PFS of 7 months (95% CI: 5.6–8.4 months) and a median OS of 12.5 months (95% CI: 9.7–15.3 months). The regimen was tolerated well, with only 11 patients (23.4%) experiencing some toxicity.

The disease control rate observed in this study is similar to the studies in which lapatinib was used in the standard doses [18–20]. In the phase III study, EGF100151, of lapatinib plus capecitabine versus capecitabine alone, the response rate was 23.7% and 13.9%, respectively. In this study, the clinical benefit rate of the combination was 29.3%, the median duration of response was 7.4 months, the median time to progression of 6.2 months and the median OS of 75 weeks [19, 20]. Similarly in the lapatinib letrozole trial, the objective response rate was 28%, the clinical benefit rate was 48% and the median PFS was 8.2 months [21, 22]. In a phase 2 trial, the response rate with lapatinib monotherapy was 24% in treatment–naïve Her2 positive metastatic patients [23]. In our study, 40.4% (*n* = 19) received Lapatinib in the third or subsequent lines which would have negatively influenced the disease control rate.

The OS was short in this study as many patients had limited access to novel anti Her2 agents and newer chemotherapeutic agents after progression on lapatinib. Remarkably, two patients (4%) had complete remission with low-dose lapatinib in the second line. Whether these deep responses to treatment can be reproduced in other larger studies remains to be seen, as genetic factors like cyclin D1 polymorphisms have been said to influence response to lapatinib [24].

There was a significantly better PFS with the use of the drug in those who had already received Trastuzumab. Previous studies have shown that the number of prior Trastuzumab-based regimens before lapatinib may influence outcomes [20]. However, this could not be explored as the numbers are small in this study.

With respect to toxicity, skin rash was the predominant complaint in contrast to diarrhea and skin rash reported in the literature [19]. Dose interruptions were required only in 6% of patients and lapatinib was discontinued due to grade 3 toxicity only in one patient (2.1%). There were no grade 4 toxicities. In the EGF100151 study of there was a 60% incidence of diarrhea in the combination arm of capecitabine with lapatinib, with 12% being grade 3 and 1% grade 4. Skin rashes were seen in 27%, with grade 3 in 1% of patients. The discontinuation rate was 13% [19]. Similarly, in the other studies, the incidence of diarrhea reported varies between 55% and 64%, with grade 3 diarrhea events contributing to around 9%–18%. The incidence of skin rashes reported were around 43%–44% and the treatment discontinuation rate due to adverse events ranged from 15% to 16% [21, 25–27].

The diarrhea and rash associated with lapatinib are believed to be due to ErbB1 inhibition in normal gut mucosa and skin [28, 29]. The occurrence and severity of diarrhea have been found to be dose dependent [29]. It has been also propounded to be related to the amount of drug in the gut after absorption, thereby decreasing the incidence of diarrhea when lapatinib is administered with food due to increased absorption [14]. This might be a reason for the reduced incidence of diarrhea seen in our study. Dermatological adverse events have been shown to predict improved outcomes, especially with early onset rash, but no such association was observed in this study [30, 31]. Furthermore, it has been found that Asian patients seem to have fewer dermatological events due to a polymorphism in intron 1 of the EGFR gene [32, 33]. This could be the reason for the lesser incidence of skin toxicities in this study.

Strategies to reduce financial toxicity are often employed in LMIC when otherwise patients may not receive the drug at all. In the case of Abiraterone acetate, the reduced dose of 250 mg/day after food was subsequently shown to have equivalent efficacy to 1,000 mg/day dosage on an empty stomach [34]. In the case of lapatinib, one small prospective study was done in China to assess the serum levels of lapatinib in fed and fasted states at the recommended dose. However, they have not studied the clinical efficacy in such states [35].

This study has several limitations besides the retrospective nature of the study and the small sample size. The patient population is very heterogenous in terms of anti-Her2 therapy received; as some patients were anti-Her2 therapy naive. The characterisation of tumour receptors at recurrence was performed only in patients with long disease-free intervals; in others, treatment was based on baseline Her2 status. We have not studied the serum levels of lapatinib achieved by this low-dose strategy in comparison to that resulting from recommended doses, and hence, it cannot be stated that both are equivalent. Asians have been found to fare better on tyrosine kinase inhibitors, but the influence of this factor on the outcomes has not been further explored in this study [36, 37].

## Conclusion

This study shows that lapatinib is effective at lower doses with lesser toxicity. Hence, the way forward is to have prospective trials to find the efficacy of lower doses of lapatinib based on pharmacokinetic data.

## Conflicts of interest

The authors declare that they have no conflict of interest.

## Funding

No research funding was obtained for this study.

## Disclosure of results

This paper was presented as a poster in the ESMO ASIA 2022.

## Institutional review

The study was approved by the institutional review board (1616/IRB-SRC/13/MCC/10-09-2021/2). Approval from the ethical committee was not required as it was retrospective in nature.

## Figures and Tables

**Table 1.** Baseline characteristics.

No.	Baseline characteristic		Number/Mean (Percentage/SD)
1	Mean Age (SD)		51 years (9.2)
2	Females		47 (100)
3	Co-morbidities – Present		20 (42.6)
4	Grade of tumour	1	2 (4.3)
		2	27 (57.4)
		3	13 (27.7)
5	ER positive tumours (HR Positive)		35 (74.5)
6	ER and PR negative (HR Negative)		12 (25.5)
7	Median Ki-67 (Range)		29.7% (5%–80%)
8	Denovo metastases		27 (57)
9	Site of metastases	Bone only	5 (10.6)
		Lymph node only	4 (8.51)
		Single visceral^a^	15 (31.9)
		Multi-visceral^b^	23 (48.9)
10	Number of lines of treatment prior to Lapatinib	One	28 (59.6)
		Two	10 (21.3)
		Three	4 (8.5)
		Four	3 (6.4)
		Five	1 (2.1)
		Six	1 (2.1)
11	Agents received prior to Lapatinib	Trastuzumab	37 (78.7)
		Taxane	41 (87.2)
		Anthracycline	11 (23.4)
		Hormonal agents^c^	23 (48.9)
		TDM1	1 (2.1)
		Others^d^	14 (29.7)

a Lung – 11, Liver – 2, Pleural deposit – 1, Brain – 1

b Mainly liver and lung, only one with brain metastases

c Tamoxifen, Letrozole and Exemestane

d Capecitabine, Gemcitabine, Carboplatin, Vinorelbine, Metronomic chemotherapy (Etoposide/Cyclophosphamide)

ER – Estrogen receptor, PR - Progesterone receptor

**Table 2.** Agent in combination with lapatinib.

No.	Agent	Number (Percentage)
1	Capecitabine	24 (51.1%)
2	Taxane	1 (2.1%)
3	Gemcitabine	2 (4.2%)
4	Tamoxifen	6 (12.8%)
5	Letrozole	4 (8.5%)
6	Exemestane	3 (6.4%)
7	Fulvestrant	3 (6.4%)
8	Nil	4 (8.5%)

**Table 3.** Outcomes.

Outcome		
Response at 3 months	CR	2 (4.3%)
	PR + SD	27 (57.5%)
	PD	17 (36.2%)
Disease control rate at 3 months		61.70%
Duration of response		4.5 months (range: 1.3 to 45.8 months)
PFS, median		7 months (95% CI: 5.6–8.4 months)
OS, median		12.5 months (95% CI: 9.7–15.3 months)

**Table 4.** Toxicities.

Type of toxicity	Grade (CTCAE v5.0)	Number of patients	Agent in combination with lapatinib
Palmar-plantar dysesthesia	3	1	Capecitabine
Palmar-plantar dysesthesia	2	5	Capecitabine
Acneiform eruptions	2	1	Capecitabine
Skin rashes	2	1	Capecitabine
Diarrhea	2	1	Nil
Thrombocytopenia	2	1	Gemcitabine
Depressive and psychosomatic symptoms	2	1	Letrozole
